# The Post-Traumatic Growth of Primary Caregivers of Patients after Liver Transplantation

**DOI:** 10.3390/healthcare10091617

**Published:** 2022-08-25

**Authors:** Ya-Hui Chen, Wei-Chen Lee, Yu-Yi Kao, Li-Chen Chen, Lun-Hui Ho, Whei-Mei Shih

**Affiliations:** 1 Department of Nursing, Chang Gung Memorial Hospital-LinKou Branch, Taoyuan 333, Taiwan; 2 Department of General Surgery, Chang Gung Memorial Hospital-LinKou Branch, Taoyuan 333, Taiwan; 3 Administrative Center Nursing Management Department, Chang Gung Memorial Hospital-LinKou Branch, Taoyuan 333, Taiwan; 4 Graduate Institute of Health Care, Chang Gung University of Science and Technology, Taoyuan 333, Taiwan

**Keywords:** stress, coping strategy, mediating effect, post-traumatic growth, caregivers

## Abstract

Liver transplantation is a very important surgery. In many cases, it involves two loved ones (receiver and donor in the same family) and causes stress and feelings of burden in family caregivers. The purpose of this study was to investigate post-traumatic growth in primary caregivers of liver transplant patients. A cross-sectional research design was adopted to recruit 84 participants. The Perceived Stress Scale, Short-Form Coping Strategies Scale, and Post-traumatic Growth Scale were used. The results revealed that the total score of perceived stress of the main caregivers of liver transplantation was 27.27 ± 6.63; problem-oriented coping and emotion-oriented coping were used as the main coping strategies, and the traumatic growth score was 42.01 ± 13.84. All three variables were significant predictors of post-traumatic growth (*F* = 13.71, *p* < 0.05), explaining 38% of the total variance. This study can help nurses understand the post-traumatic growth status and related factors of the main caregivers of liver transplant patients. It can also help caregivers understand their own perceived pressure and then take relevant care measures to reduce the degree of physical and mental load and achieve a balanced state.

## 1. Introduction

Liver failure caused by chronic liver disease and cirrhosis is a global health problem. Chronic liver disease and cirrhosis are still among the top 10 causes of death in Taiwan and the 11th leading cause of death worldwide, accounting for more than 2 million deaths globally [1,2,3]. Because there is no nerve distribution in the liver, many patients do not seek medical attention until complications such as jaundice, ascites, upper gastrointestinal bleeding, or liver cancer occur, but it is often too late. In cases where neither medical nor surgical treatment can effectively resolve the disease progression, liver transplantation becomes the only treatment option [4]. The 1–5-year survival rate after liver transplant is 74–83% [5,6,7], and the only way to fail is death.

Liver transplant surgery offers a silver lining to patients and families, but the main caregivers have to face the pain of their relatives undergoing the surgery and may even lose their family members. Therefore, liver transplant surgery represents a life-threatening medical decision and process. In Eastern traditional culture, it is the family’s responsibility to take care of sick family members. Therefore, the long-term stress of primary caregivers cannot be underestimated. Primary caregivers had more physical discomfort and more emotional distress, such as tiredness, feeling overwhelmed, anger, depression, and anxiety, than non-caregivers. Therefore, the psychosocial stress of primary caregivers may be higher than that of patients they care for [8,9,10,11,12]. However, primary caregivers may neglect their health needs when caring for patients who received liver transplants. In order not to affect their ability to perform patient care, primary caregivers use coping strategies to maintain their physical and mental health in responding to their caring stress [9,13]. In order to meet the social expectations of society and the family, caregivers adopt appropriate coping styles to adapt to the situation to avoid endangering the health of the patient and family [13,14,15]. With this response, the primary caregiver may become an invisible patient.

Studies have shown that when individuals experience significant stress, trauma, and threats to the self, it may stimulate the development of coping skills. Therefore, if one can cultivate the ability to respond positively to events, adjust to changes brought about by major events, and construct the meaning of life, it can promote the healthy development of the individual’s functioning and demonstrate resilience and post-traumatic growth (PTG) in life [16]. PTG is defined as a positive psychological change experienced as a result of struggling with highly challenging, highly stressful life circumstances involving “life-changing” psychological shifts in thinking and relating to the world and the self, which contribute to a personal process of change that is deeply meaningful [16]. Because patients have to go through a long period of time to recover, the PTG of primary caregivers of patients after liver transplantation is very important and cannot be ignored. This paper reports the findings of the first study to explore PTG in primary caregivers of patients after liver transplantation. The purpose of this study was to understand the stress, coping style, and degree of PTG of the primary caregivers of liver transplant patients, which would help them find a stable method and then develop their own positive growth to achieve a state of physical and mental balance.

### 1.1. The Status of Liver Transplantation

When the liver loses its function due to irreversible disease, it cannot be treated in traditional ways, and the only way to prolong life and have a curative effect is liver transplantation [2]. In 1963, Starzl et al. completed the first case of human liver transplantation. In 1984, Taiwan successfully completed the first liver transplant in Asia. With the maturity of surgical techniques and the development and use of anti-immune suppressants, the success rate of transplants has been greatly improved [4,17].

The source of organs for liver transplantation can be divided into cadaveric liver transplantation and living-related liver transplantation. The current Taiwan law stipulates that cadaveric liver transplantation can only be limited to blood relatives or cousinship. In 2020, the Device Donation Registration Center estimated that the total number of liver transplant recipients in Taiwan in the past decade was 5429, with 977 (18%) organ donations and 4452 (82%) living body donations [16]. In the case of limited organ sources, Living Donor Liver Transplantation (LDLT) has become the main treatment modality for patients with liver failure to prolong the life of patients with end-stage liver disease [6,18,19,20,21].

### 1.2. Concepts Related to Stress, Coping Strategies, and Post-Traumatic Growth

Stress occurs when an event resulting from the interaction between people and the environment that an individual assesses and considers creates a burden or demand that exceeds the individual’s available resources and affects the state of well-being [20]. Loss of health can be seen as a stressor or crisis that extends to the patient’s relatives and affects their quality of life at any time. The initial adaptation period of the care process, the management of sudden emergencies, and major medical decisions are all reasons for the increase in care pressure [22,23]. Coping behavior is a strategy used by individuals to change and adapt to stress when faced with a shocking situation. Therefore, the primary caregiver will adopt different coping strategies in the care process to control or reduce stressful situations. Carver, Scheier, and Weintraub (1989) used the Folkman and Lazarus theory as a basis to evaluate different ways of coping with stress and summarized the coping strategies of stress into 14 coping behaviors in three categories, including: 1. Problem-oriented coping—actively responding, seeking instrumental support, and planning. 2. Coping with emotional orientation—acceptance, emotional support, humor, positive transformation, and religious belief. 3. Dysfunction coping—disengagement, denial, distraction, self-blame, substance use (drug or alcohol), and emotional venting [24,25].

PTG is a dynamic process of the traumatic stress response, which is a positive change after resistance and struggling with highly challenging life events, and it takes time to develop [24]. Once an individual finds that their emotional tension is reduced or they are relieved of their unattainable goals, it will be transformed into a coping process of cognitive reconstruction through thinking and rumination and will continue to lead the individual to growth [26,27].

Although the primary caregiver does not experience the threat of death or the discomfort of the disease treatment, it is possible for them to experience PTG, such as inner growth and spiritual satisfaction with the patient during the treatment process. PTG is one of the important factors affecting the psychological status of the main caregiver. Negative growth may lead to the physical and mental health of the main caregiver and then affect the work of caring for patients. It can bring harmony to patients and families and create a new perspective. Therefore, the importance of assisting primary caregivers of patients after liver transplant to cope with stress and use positive coping strategies to achieve positive growth cannot be understated.

## 2. Materials and Methods

This study was accepted after being reviewed by the Human Trials Committee of the hospital (IRB 202001345B0). The cross-sectional correlation method was used to facilitate sampling. It was conducted from 1 September 2020 to 30 August 2021. The main caregivers of patients receiving liver transplants in a large teaching medical center in Taiwan were selected as the research subjects. Inclusion criteria included: (1) aged 20 or above; (2) primary caregiver of a patient receiving a liver transplant; (3) conscious and able to communicate in Mandarin or Taiwanese; (4) able to read and write; and (5) agreed to participate. It took 15–20 min to complete questionnaires. The number of samples was estimated by G-Power 3.1 (Heinrich Heine University Düsseldorf, Düsseldorf, German), F-test, linear multiple regression fixed model, R2 deviation from zero, size = 0.3, power 0.8, and *α* 0.05, and considering an attrition rate of 20%, 84 cases were collected.

The research questionnaires included: demographic questionnaires, perceived stress scales, short-form coping strategies scales, and post-traumatic growth scales.

(1) Demographic questionnaire: It was prepared with reference to the relevant literature and included: gender, age, education level, marital status, religious beliefs, pre-transplant occupation of care, annual family income, length of care, and relationship to liver transplant recipients.
(2) Perceived Stress Scale (PSS): This study used the Perceived Stress Scale developed by Cohen et al. In 2005, Dr. Li-Chuan Chu was authorized to translate it into a Chinese version and used a self-reported method to measure the degree of stress in an individual’s life in the past month. A five-point Likert scale scoring method was used with the five options “never”, “occasionally”, “sometimes”, “often”, and “always”, giving 0, 1, 2, 3, and 4 points. There were 14 questions in total, of which 7 were positive questions, and the other 7 were reverse questions. First of all, the 7 positive questions must be scored in the reverse direction on the scale, and all scores must be added together. The total score is 0–56 points. The higher the score, the higher the perceived stress intensity of the subject. The scale reliability based on Cronbach’s α value was 0.85 in other studies and 0.71 in this study, which is acceptable [28].
(3) Brief Coping Strategies Scale (Brief COPE): Based on the Brief COPE adapted by Carver in 1997, strategies were divided into problem coping strategies, emotional coping strategies, and dysfunctional coping strategies. A total of 14 coping strategies were measured, including active coping, emotional support, instrumental support, positive reinterpretation, planning, acceptance, denial, substance use, self-distraction, catharsis, behavioral avoidance, humor, religion, and self-blame. Each coping strategy consisted of 2 questions, with a total of 28 questions, and its Cronbach’s α coefficient was between 0.50 and 0.90. The score was on a four-point scale, with the four options “never”, “occasionally”, “sometimes”, and “often”, giving 1, 2, 3, and 4 points, respectively. The higher the behavioral characteristics of this dimension, the better the coping has. Huei-Jia Tzeng, Chang-Chu Ho, and Ming-Chang Tsai (2010) conducted Brief COPE in Chinese culture. After obtaining the questionnaire from the original author, the content was scored and revised according to the relevance, correctness, and wording appropriateness of the research topic questionnaire. After synthesizing expert opinions, the content validity was completed. The internal consistency reliability of the scale was Cronbach’s α of 0.86, and the Cronbach’s α in this study was 0.84 [29].
(4) Posttraumatic Growth Inventory: The questionnaire was developed by Tedeschi and Calhoun in 1996, and Ho et al. (2004) translated it into the Chinese version. The internal consistency reliability based on Cronbach’s α of the Chinese version was 0.86, with a total of 4 subscales: self, spirituality, life direction, and interpersonal. It is based on a six-point scale with “no change at all” “, “Very small change”, “Small change”, “Medium change”, “Obvious change”, and “Very large change”, giving 0, 1, 2, 3, 4, and 5. A score greater than or equal to three was considered moderate or above growth. In this study, Cronbach’s α was 0.91 [30].

The data were statistically analyzed with SPSS (IBM, Armonk, NY, USA) for the Window 23.0 Chinese version software package. The statistical significance level was set to *p* < 0.05. The statistical methods included percentage, mean, standard deviation, etc., for descriptive statistics, and standardized scores were used. Independent sample t-test, one-way analysis of variance, Pearson product-difference correlation analysis, and stepwise multiple regression analysis were used for inferential statistics. Before performing the stepwise multiple regression analysis, the independent variables were tested for collinearity, independence and normality, and specificity. Dysfunctional coping strategies, etc., were listed as independent variables, post-traumatic growth was the dependent variable, and a stepwise multiple regression analysis was performed.

**Figure 1:**
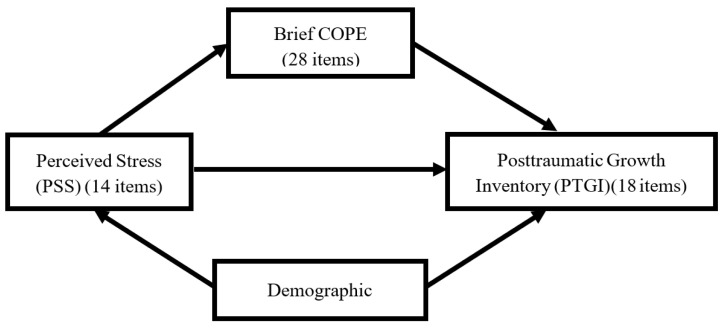
The structure of the research design.

The structure of the research design is illustrated below (Figure 1).

## 3. Results

A total of 84 primary caregivers of patients who received a liver transplant participated in this study (Table 1).

### 3.1. Demographic Variables of Caregivers

More than half of the caregivers were women, with 58 (69.0%). The average age was 45.32 years (±13.02), with the oldest being 74 years old and the youngest being 20 years old. Among them, the majority were 20 to 49 years old, with a total of 48 (57.1%) participants; 42 (50.0%) participants completed high school or less; 63 (75.0%) participants were married; 61 (72.6%) participants had a religion; 39 main caregivers (46.4%) were the patient’s spouse; 35 (41.7%) participants had an annual household income of less than NTD 600,000 (approximately USD 21,000); 59 (70.2%) participants had no chronic diseases; 70 (83.3%) participants spent less than one year in caregiving; and 55 (65.5%) participants received living donor transplants.

### 3.2. Analysis of Care Stress, Coping Strategies, and Post-Traumatic Growth among Primary Caregivers

The primary caregivers’ PSS was 27.27 ± 6.63 points. Among the coping strategies, problem-oriented coping was 20.51 ± 3.34, emotion-oriented coping was 31.02 ± 4.96, and dysfunctional coping was 23.07 ± 4.99. Comparing the three coping results, the use of problem-oriented coping strategies was higher than that of emotion-oriented coping strategies and dysfunctional coping. As for traumatic growth, the score was 42.01 ± 13.84 points. The order of traumatic growth scores from highest to lowest was life direction, interpersonal, self, and spirituality (Table 2).

### 3.3. Differences in Primary Caregiver Demographic Variables and Post-Traumatic Growth

Demographic variables of the primary caregiver of liver transplant patients and the percentage difference in the caregivers’ PTG scores (Table 1) showed that there were significant differences in gender and the primary caregiver role among caregivers and PTG (*p* < 0.05). The PTG of spouses was more significant than that of children and parents.

The results of this study (Table 3) found that there was a significant correlation between women and PTG (*r* = 0.485, *p* < 0.01), ego (*r* = 0.438, *p* < 0.01), interpersonal (*r* = 0.433, *p* < 0.01), life direction (*r* = 0.300, *p* < 0.01), and spirituality (*r* = 0.467, *p* < 0.01). Children as caregivers were significantly negative correlated with PTG (*r* = −0.218, *p* < 0.05), ego (*r* = −0.254, *p* < 0.05), and life direction (*r* = −0.240, *p* < 0.05). There was a significant correlation between perceived stress and PTG (*r* = 0.338, *p* < 0.01), ego (*r* = 0.365, *p* < 0.01), interpersonal (*r* = 0.261, *p* < 0.01), and life direction (*r* = 0.282, *p* < 0.01). The higher the perceived stress, the higher the PTG.

There was a significant correlation between problem-oriented coping and overall PTG (*r* = 0.369, *p* < 0.01), ego (*r* = 0.362, *p* < 0.01), interpersonal (*r* = 0.315, *p* < 0.01), life direction (*r* = 0.274, *p* < 0.05), and spirituality (*r* = 0.281, *p* < 0.01). The more problem-oriented coping used, the more significant the PTG. Emotional-oriented coping was associated with overall PTG (*r* = 0.449, *p* < 0.01), ego (*r* = 0.384, *p* < 0.01), interpersonal (*r* = 0.431, *p* < 0.01), life direction (*r* = 0.262, *p* < 0.05), and spirituality (*r* = 0.454, *p* < 0.01), and the correlations were significant. The more emotional-oriented coping used, the more significant the PTG, and the PTG was higher in emotional-oriented coping than in problem-oriented coping.

### 3.4. The Mediating Effect of Coping Strategies on Perceived Stress and Post-Traumatic Growth

In this study, PROCESS v3.5 was used to test the mediating effect of the Sobel test to test the influence of coping strategies on perceived stress and PTG. The three coping styles were used as mediating variables (M), perceived stress was the independent variable (X), and PTG was the dependent variable (Y) when testing the mediating effect of the coping style on perceived stress and PTG. The results showed that emotional response to PTG had a Bootstrapping 95% CI of [0.08, 0.65] and Z value of 3.35 (*p* < 0.001), indicating that emotion-oriented coping has a mediating effect on coping with PTG (Table 4).

### 3.5. Factors Affecting the Post-Traumatic Growth of the Primary Caregivers of Liver Transplant Patients

With PTG as a dependent variable, gender, primary caregiver relationship, perceived stress, problem-oriented coping, and emotional-oriented coping had statistically significant differences or correlations as independent variables. Among them, the relationship between gender and primary caregiver is a categorical variable, and for the regression analysis, a stepwise multiple regression statistical analysis method was carried out. The results revealed (Table 5) that the primary predictor of the PTG of the main caregivers of LT patients was gender, which explained 23% of the variance; other sequential variables were perceived pressure, care relationship for children, and emotional coping, which explained 38% of the variance, showing that this model had a predictive ability for PTG.

## 4. Discussion

This study found that the PTG of the primary caregivers of liver transplant patients was correlated with gender, children as caregivers, perceived stress, problem-oriented coping strategy, and emotional-oriented coping strategy, which also had a mediating effect. Caregivers showed moderate PTG, with a mean of 42.01 ± 13.84 points (0–75 points), and women had statistically significantly more PTG than men (*p* < 0.05). Several researchers [31,32,33] reported that post-traumatic growth was related to gender in the same way, but the results are different from Ho’s study [32], which may be due to differences in the attributes of the objects of care of the research subjects. In the Eastern tradition, gender role expectations and family cultural concepts advocate that “men should be outside, women should be inside”. Married women’s care responsibilities are attributed to gender identity and moral development, as well as social and cultural structures. Even though Taiwan has undergone social, economic, and demographic changes, and the family care model is different from the past, the constant is that the role of care is still mostly held by women in the family, and the care recipients are usually children, spouses, and parents [34].

In this study, the gender distribution of the main caregivers was dominated by female spouses, which is similar to most previous studies [34,35,36,37]. The primary caregivers had moderate perceived stress of 27.27 ± 6.63 (0–56 points) during the caregiving process. Regardless of women’s caring ability, in Chinese culture, women are given care responsibilities, thus increasing their care pressure. Seventy (83.3%) caregivers spent less than one year in caregiving, with an average of 5.71 ± 5.68 months. This study found that 57 (67.9%) primary caregivers chose to suspend work to focus on caring for the liver transplant patient in order to concentrate on providing proper care. In the process of approaching the main caregivers, they told the researchers that because they had to take care of the patient alone, it affected their sleep and health. However, in the face of progress and changes in the patient’s condition, at the same time, they also had to worry about financial resources and the condition of other family members, so they often perceived the existence of stress. Stress in caregivers may lead to poor sleep quality and high-risk diseases, such as cardiovascular disease, cancer, metabolic disease, depression, and dementia [36,37,38]. Therefore, medical personnel need to assist and care for caregivers to help them deal with and use appropriate coping skills to reduce the perceived stress caused by the care process [39].

Studies have shown that PTG varies among children as primary caregivers. Some showed negative correlations, while others showed lower negative effects and higher life satisfaction [40,41]. These results may be related to the length of time that children are involved in care. The longer the care, the better the PTG. Whether or not the primary caregiver is actively coping affects their post-traumatic growth and predictability. Most people use a mix of coping strategies when they are under stress. When an individual is faced with an unchangeable situation, he/she mostly adopts an emotion-oriented coping strategy, while for a changeable situation, he/she chooses to use a problem-oriented approach, which is consistent with the results of this study [22,42,43].

## 5. Conclusions

This study presented important factors affecting the PTG of primary caregivers of liver transplant patients. Stepwise regression analysis concluded that gender, perceived stress, and the use of emotion-oriented coping strategies were important predictors of main caregivers’ PTG. Healthcare providers should take these factors into consideration when caring for primary caregivers of liver transplant patients.

If the positive coping ability of the primary caregiver can be improved and enforced, changes after their adjustment can give them a new understanding and allow them to construct the meaning of life, which will promote the individual’s resilience in life. 

In conclusion, caregivers should be actively included in the scope of assistance in clinical medical care to provide better and complete care.

## Figures and Tables

**Table 1:** Analysis of basic attributes and post-traumatic growth of major family caregivers of liver transplantation (n = 84).

Item	*n*	%	*Mean*	*SD*	*t/F*	Scheffe
**Sex**					5.03 ***	
Male	26	31.0	32.04	14.38		
Female	58	69.0	46.48	11.06		
**Age**			45.32	13.02	−1.55	
20–49	48	57.1	40.00	13.83		
≥50	36	42.9	44.69	13.57		
**Education**					0.78	
≤High school	42	50.0	43.19	13.41		
≥College	42	50.0	40.83	14.32		
**Marriage**					−1.43	
Single	21	25.0	38.29	13.06		
Married	63	75.0	43.25	13.96		
**Religion**					0.30	
Yes	61	72.6	42.30	14.54		
No	23	27.4	41.26	12.04		
**Caregiver**					5.40 **	③ > ①,②
① Spouse	39	46.4	44.85	12.345		
② Children	22	26.2	32.82	15.083		
③ Parents	12	14.3	48.33	9.365		
④ Others	11	13.1	43.45	12.972		
**Occupation before caring**					−0.58	
Yes	57	67.9	41.40	13.86		
No	27	32.1	43.30	13.967		
**Household income**					2.08	
≤USD 21,000	35	41.7	39.77	15.564		
≥USD 21,000 < 28,000	32	38.1	41.34	14.086		
③ ≥ 28,000	17	20.2	47.88	6.744		
**Chronic disease**					−2.01	
Yes	25	29.8	37.44	14.73		
No	59	70.2	43.95	13.09		
**Caring time**			5.71	5.68	−1.01	
0–12 months	70	83.3	41.33	13.77		
≥12 months	14	16.7	45.43	14.19		
**Organ source**					0.01	
Relatives	55	65.5	42.02	13.79		
Cadaveric	29	34.5	42.00	14.17		

Note. ** = *p* < 0.01. *** = *p* < 0.001.

**Table 2:** Analysis of perceived stress, coping strategies, and post-traumatic growth of primary caregivers.

Item	Range	*Mean*	*SD*	Standardized Score ^a^	Order
**Perception stress**	0–56	27.27	6.63		
**Coping strategies**					
Problem-oriented	6–24	20.51	3.34	85.46	1
Emotion-oriented	10–40	31.02	4.96	77.55	2
Dysfunctional	12–48	23.07	4.99	48.06	3
**Post-traumatic growth**	0–75	42.01	13.84		
Self	0–35	19.70	6.57	56.29	3
Interpersonal	0–15	8.83	3.21	58.87	2
Life direction	0–10	5.98	2.54	59.80	1
Spirituality	0–15	7.50	3.61	50.00	4

Note. Standardized score formula ^a^ = X − μ/σ.

**Table 3:** Caregivers’ perceptions of stress, coping strategies, and post-traumatic growth.

Item	Female	Caregiver- Children	Perceived Stress	Problem- Oriented Coping	Emotion- Oriented Coping	Dysfunctional Coping
Post-traumatic growth	0.485 *	−0.218 *	0.338 **	0.369 **	0.449 **	0.041
Self	0.438 **	−0.254 *	0.365 **	0.362 **	0.384 **	0.056
Interpersonal	0.433 **	−0.148	0.261 **	0.315 **	0.431 **	0.011
Life direction	0.300 **	−0.240 *	0.282 **	0.274 *	0.262 *	−0.066
Spirituality	0.467 **	−0.073	0.198	0.281 **	0.454 **	0.092

Note. * = *p* < 0.05. ** = *p* < 0.01.

**Table 4:** Testing the mediating effect of coping strategies for perceived stress on post-traumatic growth.

Item	Effect	Standardized Estimate	Bootstrapping 95% CI	Z
Total indirect effect	0.30	0.19	[−0.02, 0.70]	
Problem-oriented	−0.01	0.20	[−0.37, 0.42]	2.16
Emotion-oriented	0.35	0.15	[0.08, 0.65]	3.35 ***
Dysfunctional-oriented	−0.04	0.06	[−0.17, 0.06]	0.10

Note. *** = *p* < 0.001.

**Table 5:** Stepwise regression analysis of post-traumatic growth of primary caregivers of liver transplant patients (*n* = 84).

Model	Variables	R2	Adjusted *R2*	*F*	*B*	S.E.	*β*	*t*
1	Gender	0.24	0.23	25.29	14.44	2.87	0.49	5.03 ***
2	Perceived stress	0.32	0.30	18.68	0.59	0.19	0.29	3.08 **
3	Caregiver-children	0.36	0.34	15.21	−7.34	3.00	−0.24	−2.44 *
4	Emotion-oriented coping	0.41	0.38	13.71	0.72	0.29	0.26	2.50 *

Note. * = *p* < 0.05. ** = *p* < 0.01. *** = *p* < 0.001.

## Data Availability

The datasets used and/or analyzed during the current study are available from the corresponding author upon reasonable request.
